# Neural patterns elicited by lexical processing in adolescents with specific language impairment: support for the procedural deficit hypothesis?

**DOI:** 10.1186/s11689-022-09419-z

**Published:** 2022-03-19

**Authors:** Julia L. Evans, Mandy J. Maguire, Marisa L. Sizemore

**Affiliations:** 1 School of Behavioral and Brain Sciences, University of Texas at Dallas, Dallas, TX USA; 2 Mendocino County Office of Education, Ukiah, CA USA

**Keywords:** Specific language impairment (SLI), Developmental language disorder (DLD), Procedural deficit hypothesis, Procedural memory, Semantic processing, Spoken word processing, Imageability, Event-related potentials, N400, N700

## Abstract

**Background:**

Deficits in procedural memory have been proposed to account for the language deficits in specific language impairment (SLI). A key aspect of the procedural deficit hypothesis (PDH) account of SLI is that declarative memory is intact and functions as a *compensatory* mechanism in the acquisition of language in individuals with SLI. The current study examined the neural correlates of lexical-phonological and lexical-semantic processing with respect to these predictions in a group of adolescents with SLI with procedural memory impairment and a group of chronologically age-matched (CA) normal controls.

**Methods:**

Participants completed tasks designed to measure procedural and declarative memory and two ERP tasks designed to assess lexical-semantic and lexical-phonological processing in the auditory modality. Procedural memory was assessed using a statistical learning task. Lexical-semantic processing was assessed using a sentence judgment task modulating semantic congruency and lexical-phonological processing was assessed using a word/nonword decision task modulating word frequency. Behavioral performance on the tasks, mean amplitude of the cortical response, and animated topographs were examined.

**Results:**

Performance on the statistical word-learning task was at chance for the adolescents with SLI, whereas declarative memory was no different from the CA controls. Behavioral accuracy on the lexical-semantic task was the same for the adolescents with SLI and CA controls but accuracy on the lexical-phonological task was significantly poorer for the adolescents with SLI as compared to the CA controls. An N400 component was elicited in response to semantic congruency on the lexical-semantic task for both groups but differences were noted in both the location and time course of the cortical response for the SLI and CA groups. An N400 component was elicited by word frequency on the lexical-phonological task for the CA controls not for the adolescents with SLI. In contrast, post hoc analysis revealed a cortical response based on imageability for the adolescents with SLI, but not CA controls. Statistical word learning was significantly correlated with speed of processing on the lexical decision task for the CA controls but not for the adolescents with SLI. In contrast, statistical word learning ability was not correlated with the modulation of the N400 on either task for either group.

**Conclusion:**

The behavioral data suggests intact semantic conceptual knowledge, but impaired lexical phonological processing for the adolescents with SLI, consistent with the PDH. The pattern of cortical activation in response to semantic congruency and word frequency suggests, however, that the processing of lexical-semantic and lexical-phonological information by adolescents with a history of SLI may be supported by both overlapping and nonoverlapping neural generators to those of CA controls, and a greater reliance on declarative memory strategies. Taken together, the findings from this study suggest that the underlying representations of words in the lexicons of adolescents with a history of SLI may differ qualitatively from those of their typical peers, but these differences may only be evident when behavioral data and neural cortical patterns of activation are examined together.

## Introduction

Specific language impairment (SLI), also known as developmental language disorder (DLD), is a neurogenetic developmental language disorder of unknown etiology characterized by failure to master spoken and written language comprehension and production. Although numbers vary slightly, in the United States, SLI occurs in ~ 7% of English-speaking school-aged children [1]. Various accounts have been proposed for the SLI behavioral deficit profile. These include domain-specific accounts that posit that the disorder is the result of deficits in the grammar system and domain-general accounts that posit that the disorder is secondary to impaired perceptual and/or cognitive processing deficits [2, 3]. Ullman and colleagues have proposed an alternative account that directly links the behavioral deficit profile to distinct brain circuits. Known as the procedural deficit hypothesis (PDH), the account posits that children with SLI have abnormalities in the brain circuitry of the procedural memory system, which in turn leads to impaired procedural memory function and the subsequent impairment in the acquisition and use of those aspects of language hypothesized to be supported by this system. In contrast, the PDH account posits that the circuitry of the declarative memory system is largely intact in children with SLI, leaving those aspects of language hypothesized to be supported by the declarative memory system unimpaired in these children [4–6].

Children with SLI consistently demonstrate poor performance on the traditional tasks designed to assess procedural memory (i.e., Serial Recall Tasks (SRT)). For example, a meta-analysis of studies using STR tasks reveals a consistent pattern of poor procedural memory in children with SLI [7, 8]. Similarly, a meta-analysis by Obeid, et al. [9] that included a broader range of procedural learning tasks (i.e., SRT, artificial grammar learning, probabilistic classification, etc.) also found that children with SLI showed significant impairments in procedural learning as compared to controls regardless of task modality (e.g., visual versus auditory). Although the focus of research within the PDH theoretical framework has been the identification of procedural memory deficits in SLI and their link to morphology and syntax deficits in these children [8–10], the PDH also predicts deficits for some but not all aspects of lexical processing as well. Specifically, the PDH posits that because procedural memory supports the concatenation and computation of the sequential information, deficits are predicted at the lexical-phonological level which subsequently impacts spoken word recognition, lexical retrieval, and sensitivity to word frequency effects (i.e., faster processing of high-frequency versus low-frequency words) [6].

Children with SLI are slower and less accurate in learning new words as compared to their peers [11–13], and once acquired, the lexical-phonological representations of words in the lexicons of children with SLI are organized differently from those of normal language peers [14–16]. Unlike their typical peers who are faster at accessing high as compared to low-frequency words, children with SLI are significantly slower at accessing both high- and low-frequency words from their lexicon [17], are slower to recognize spoken words, and are more vulnerable to lexical cohort interference than their peers [18, 19] due to poorly specified lexical-phonological representations [20].

Although much of the research on procedural memory deficits in children with SLI has focused on their poor performance on serial recall tasks (SRT) and artificial grammar learning tasks, children with SLI also have significant difficulty holding the sequential order of phonemes and syllables within the speech stream in memory and implicitly tracking and computing the probabilities of adjacent sound sequences. Typically referred to as “statistical word learning” (SWL), this ability to track and compute sequential statistics in the stream of speech—such as the transitional probability across phonemes—to discover word boundaries within a stream of speech relies heavily on the procedural memory system and has been linked to word learning and vocabulary acquisition in typically developing children [21]. Further, behavioral studies of statistical learning task in children with SLI have shown that their performance on statistical learning tasks is consistently poorer than that of their typical peers and is linked both to deficits in receptive and expressive vocabulary and spoken word recognition in both monolingual and bilingual children with SLI [19, 22, 23].

Because the circuitry of the declarative memory system is hypothesized to be largely intact in SLI, the PDH account predicts that those aspects of language supported by declarative memory also are intact in these children. The declarative memory system is involved in the encoding of episodic information, real-world knowledge, personal experiences, and the arbitrary, idiosyncratic lexical-semantic aspects of word knowledge. Traditionally, studies of verbal declarative memory in children with SLI have used word list-learning tasks where they hear lists of words and are asked to recall the words in the lists. Children with SLI consistently perform below their peers on these tasks; however, as Lum and colleagues [24] note, a confound in these tasks is the concomitant verbal working memory demands of the task coupled with the working memory deficits consistently seen in children with DLD, in particular deficits in the ability to hold auditory information in memory [25]. In contrast, children with SLI do perform within normal limits on standardized measures of receptive and expressive vocabulary; however, experimental studies suggest that lexical-semantic representations of children with SLI may not be as richly represented and specified as compared to those of their typically developing peers [19, 26–28].

Ullman and colleagues argue that children with SLI may *appear* to have lexical-semantic deficits when examined under experiment conditions where they are forced to rely on their impaired procedural memory, but under experimental conditions where they can rely on declarative memory system, their intact semantic conceptual knowledge will be evident [4]. Ullman and colleagues also argue that because children with SLI may rely on declarative memory as a compensatory strategy in their acquisition of language, there should be residual evidence of this compensation in the neural underpinnings of their language processing [5]. Electrophysiological measures, which do not require a behavioral response, provide a means of studying the possible compensatory role of declarative memory in these children. EEG studies of adolescents with a history of SLI show that their behavioral performance appears to “resemble” that of typical developing peers, but that the underlying neural circuits that are engaged by these adolescents with SLI differ qualitatively from those of their typically developing peers [29, 30] and a small number of studies suggest the possible compensatory reliance on declarative memory networks during language processing in adolescents with SLI (e.g., [31, 32]). For example, Fonteneau & van der Lely [31] observed that syntactic violations elicited the standard early left anterior negativity (ELAN) in normal language controls, but not in adolescents with SLI. Instead, in the adolescents with SLI, syntactic violations elicited an N400 response in the right posterior regions—a cortical response traditionally link to semantic processing in typically developing individuals. Similarly, in one of the only anatomically constrained magnetoencephalography (aMEG) studies of adolescents with SLI, Brown and colleagues [32] also observed that behavioral accuracy on a semantic classification task was the same for typically developing controls and an adolescent with SLI with documented procedural memory deficits. In contrast to his “normal” behavioral performance, the dynamic functional organization of his cortical activation was atypically right lateralized in a manner characteristic of right hemisphere specialization for nonverbal concrete, conceptual representations, in contrast to the more typical left temporal-parietal pattern of activation evident in the normal controls [32].

### Current study

Thus far no studies have directly examined procedural and declarative memory and the electrophysiological response to lexical-semantic and lexical-phonological information in adolescents with and without SLI. In the current study, we focus on the EEG components linked to the processing of both lexical-semantic and lexical-phonological contexts. Initially shown to index lexical-semantic access and context [33], the electrophysiological response to words during the “N400” time window is affected not only by semantic context but also by word frequency. For example, for spoken words, independent effects are evident for word-based variables which include temporally and spatially widespread effects on semantic expectancy, word frequency, phonological neighborhood density, concreteness, and imageability. In one of the first studies to examine the developmental change in the role of semantic context on lexical-semantic processing, Holcomb and colleagues [34] showed that semantic expectancy effects to auditory sentences in adolescents were associated with an increased ERP negativity to semantically anomalous endings between ~ 500 and 800 ms being largest in the right hemisphere over the central and parietal regions. More recent studies of spoken word recognition show that word frequency is associated with increased ERP negativity to lower word frequency peaking ~ 500 ms and persisting through 900 ms primarily over frontal and central regions [35]. ERP studies also show that word imageability contributes to the modulation of real words in the N700 time window [36]. For example, an N700 even-related potential for concrete versus abstract words—semantic richness—has been linked to the processing of mental imagery [35, 37] with imageability and concreteness being linked to both early (500–700 ms), and late N700 (500–700 ms) time windows [34, 36]. Although ERP studies of lexical processing in children with SLI have yielded mixed results, they show that children with SLI evidence different patterns of cortical activity in response to lexical-phonological and lexical-semantic information [30, 38] making these components ideally suited to investigate potentially atypical cortical responses during lexical-semantic and lexical-phonological processing in adolescents with SLI.

The PDH account leads to several predictions. Adolescents with a history of SLI should evidence intact declarative memory if lexical encoding demands are removed from tasks measuring declarative memory. Further, if declarative memory is intact, then real-world knowledge also should be intact in these adolescents with SLI and should be evident in both their behavioral accuracy and cortical response to task which allow them to rely on declarative memory and tap into intact lexical-semantic representations. In contrast, these same adolescents with SLI should evident poor procedural memory abilities, and slower less accurate processing of high-frequency versus low-frequency words as compared to typical controls on lexical processing tasks that force them to rely on procedural memory and lexical-phonological information. This poor behavioral performance should be coupled to an atypical cortical response to word-frequency on a lexical decision task.

## Methods

### Participants

The participants consisted of a total of 35 adolescents ages (10;5–18;11). Data from seven participants were excluded because they did not fit our criteria (3 were left-handed, 4 did not meet the criteria for SLI and/or normal language controls). No participants were excluded because of excessive artifacts in their electroencephalography (EEG) data. Data from 28 adolescents (ages 11;11–18;11) are reported, including 14 children with SLI (females = 6), and 14 typically developing, chronological age-matched (CA) controls (females = 5). All participants were right-handed, native speakers of English, had normal or corrected-to-normal vision, and had no history of neuro-developmental, emotional, or behavioral disturbances, motor deficits, frank neurological signs, seizure disorders, or use of medication to control seizures.

### Standardized assessment

All of the participants were drawn from the SLI and TD subgroups of the San Diego Project in Cognitive and Neural Development (PCND)—initiated in 1990 (PI, Stiles). The PCND project was a longitudinal project initiated in 1990 to investigate neurodevelopmental disorders in children. The participants in the PCND-SLI group were required to meet the following criteria: (1) documented language impairment; (2) receiving speech and language services; (3) performance IQ (PIQ) of 80 or higher on the WISC-R, WPPSI, or Leiter non-verbal measures; (4) no major neurological abnormalities (determined by a neurological examination); (5) expressive language composite score 1.5 or more standard deviations below the mean on the CELF-R (Semel, Wigg, & Secord, 1987); and (6) absence of known developmental disorders such as intellectual disabilities or autism. The individuals in the PCND-TD subgroup were required to meet the same criteria with the exception that performance on standardized tests of intelligence, language, and academic functioning was within normal limits. Oral and speech motor abilities were within normal limits based upon direct observation by a speech-language pathologist, and hearing was within normal limits based upon American Speech-Language-Hearing Association guidelines (e.g., 20 dB HL at .5, 1, 2, and 4 kHz) [39].

The participant’s inclusion in the SLI and TD groups based on their initial PCND classification criteria was maintained in the current study. To confirm SLI and TD group membership, for the current study, all participants completed a battery of standardized tests in addition to the experimental protocols. This follow-up standardized testing was completed by a certified speech-language pathologist and included: (1) Leiter-R Brief nonverbal IQ (NVIQ, Leiter International Performance Scale-Revised [40];), (2) Formulated Sentences and Recalling Sentences subtests of the Clinical Evaluation of Language Fundamentals, Fourth Edition (CELF-4 [41];), (3) Nonliteral Language and Meaning from Context subtests from Comprehensive Assessment of Spoken Language (CASL [42];), and (4) Comprehensive Receptive and Expressive Vocabulary Test, Second Edition (CREVT-2 [43];). All participants continued to have normal nonverbal intelligence. The language skills for the adolescents with a history of SLI continued to be significantly below those of their age-matched peers with typical language development (Table 1). Consistent with research indicating that there is often language growth for children with a history of SLI between ages 7 and 17, the Core Language Scores for 12:14 of the participants with SLI continued to fall below 1.5 SD of age expectations, and test scaled scores for all 14:14 of the participants with SLI on the Recalling Sentences subtest of the CELF-4 continued to fall at or below -2 SD below age expectations. All of the participants also met the following inclusion/exclusion criteria: (a) passed a pure-tone audiometric screening at 20-dB HL at 500, 1000, 2000, and 4000 Hz (American Speech-Language-Hearing Association, 1997); (b) normal or corrected vision according to parent report; (c) normal oral and speech motor ability as assessed by a certified speech-language pathologist; (d) speech intelligibility of 95% or higher based on spontaneous language sample; (e) right-handed; and (f) from monolingual English-speaking home.

**Table 1:** Standardized measures for specific language impairment (SLI) and chronological age-matched (CA) controls

	SLI			CA			
	Mean	SD	Range	Mean	SD	Range	
Age in months	182	26	140–220	172	22	142–219	*p* = .23
Leiter-R: Nonverbal IQ ^a^	104	15	82–127	113	10	100–127	*p* = .05
CELF-4 ^b^ Core Language Score	79.75	14	52–99	117.7	7	108–132	*p* < .00
CELF-4^b^ Formulated Sentences	6.9	3	2–11	13.2	1	10–15	*p* < .00
CELF-4^b^ Recalling Sentences	2.6	2	1–6	11.9	2	8–14	*p* < .00
CASL^c^ Nonliteral Language	74.5	10	52–92	102.8	10	81–118	*p* < .00
CASL^c^ Meaning from Context	77.5	12	60–93	110.7	13	88–129	*p* < .00
CREVT^d^ Expressive	81.7	10	63–100	105.1	9	90–115	*p* < .00
CREVT^d^ Receptive	85	12	66–101	107.1	11	80–118	*p* < .00

^a^Leiter-R (International Performance Scale-Revised, [40])

^b^Clinical Evaluation of Language Fundamentals-4 (CELF-4 [41];)

^c^Comprehensive Assessment of Spoken Language (CASL [42];)

^d^Comprehensive Receptive Expressive Vocabulary Test-2 (CREVT [43];)

### General procedure

The standardized assessment measures and statistical learning tasks were completed as part of a larger ongoing project. As part of the project, parents also filled out a background questionnaire that included questions pertaining to speech, language, hearing, neurological, and medical history. Following participation in the larger project, participants returned to complete the two EEG tasks, completing ½ of the trials for each task in two consecutive visits over a 2-week time period. Prior to participation in the studies, written consent/assent was obtained from participants and their parents/guardian in accordance with the Institutional Review Board at San Diego State University.

### Behavioral measures

#### Statistical learning

Although the research investigating procedural sequential memory in SLI has relied on perceptual-motor learning tasks (i.e., serial reaction time tasks (SRT), [44]), because the focus of this study was spoken language processing, the adolescent’s procedural memory was assessed using the statistical word learning task in Evans et al. [22]. Adolescents listened to the exposure language for a total of 21 min. Following the exposure phase, participants completed a 2 alternative forced-choice task where they heard pairs of tri-syllables (a “word” paired with a nonword) where none of the nonwords occurred in the speech stream. The exposure language consists of 12 CV syllables comprising seven consonants and vowels (p, t, d. b, a, I, and u). The CV pairs were combined into six trisyllabic “words” (*dutaba*, *tutibu*, *pidabu*, *patubi*, *bupada*, and *babupu*). The language was constructed to ensure that the transitional probabilities between syllables within the words were higher than the transitional probabilities between syllables across word boundaries where the within-word transitional probabilities ranged from 0.37–1.0 and the across word boundary transitional probabilities ranged from 0.1 to 0.2. The exposure language was constructed with the constraint that the same word could not occur twice in a row, resulting in a 4536-syllable, continuous speech stream having no acoustic cues to word boundary, no prosodic cues, and no pauses between or within the words. For the test phase, adolescents heard pairs of words+nonwords and were asked to choose the “word” in each pair that sounded more like the speech they heard during the exposure language. To ensure that the participants understood the task, prior to completing the test trials, adolescents completed a series of practice trials containing word-nonword pairs derived from words in English (e.g., *com-pu-ter* vs *pu-ter-com*). Following the practice trials, the adolescents were presented with the 36 test pairs where the “words” were exhaustively paired with each nonword foil. All of the adolescents were able to successfully complete the practice trials and no participant was excluded from the study due an inability to understand the task.

#### Declarative memory

In the current study, we assessed children’s real world conceptual knowledge as a *stand-in* for their declarative memory. Our reasoning was that, because the declarative memory system supports both episodic memory—the storage and recall of memories of specific personal experiences and past events (e.g., an individual’s personal experience with cats), *and* semantic memory—general world knowledge such as facts, ideas, meanings, and concepts (e.g., information about what cats are), if children’s declarative memory and nonverbal IQ were both intact, then their conceptual knowledge of the real-world properties of common objects would also be intact. To assess children’s general conceptual knowledge, we used the True/False portion of the Competing Language Processing Task (CLPT, [45]). Adolescents listened to groups of 2, 3, 4, 5, and 6 sentences, responding “true” or “false” to the truth value of each sentence. After each group of sentences, participants were asked to recall the last word of each sentence in the group. Thus, the truth of each statement must be determined while the last word of each sentence is held in working memory. Because the True/False response portion of the CLPT is designed to prevent children from focusing exclusively on the word-recall portion of the task, the sentence comprehension portion of the task is designed to ensure a high degree of comprehension success. The sentences are constructed to control for length and difficulty, with each sentence consisting of three words familiar to 6;0 (subject-verb-object, subject-verb-modifier, subject-auxiliary- main verb). Half of the sentences in each group were true and half were false (i.e., *Trees have leaves*, *Trains can fly*, *Pumpkins are purple*, *Buses have wheels*). Children with SLI consistently demonstrate high-performance levels on the True/False sentence comprehension portion of the task [46, 47]. For the purposes of this study, adolescents’ accuracy on the True/False portion of the CLPT was used as a measure of declarative memory. Adolescents listened to a total of 42 sentences. Participants completed a series of practice trials prior to completion of the task and no participant was excluded due to failure to complete all of the practice trials.

### Electrophysiological tasks

#### EEG procedures

Participants completed two EEG tasks designed to examine cortical activation patterns in response to lexical-semantic and lexical-phonological processing respectively. For both tasks, participants sat approximately 65 cm from a 46-cm computer screen in an electrically shielded, sound-attenuated room with low lighting, in a comfortable chair. To reduce movement artifacts, adolescents were seated comfortably in a position that required only the lifting of their thumbs to respond, with the button box placed in their laps and their thumbs resting on the buttons. Stimuli were presented binaurally through loudspeakers at ~ 65-dB SPL. During the tasks, participants were instructed to keep their eyes focused on a fixation cross in the middle of the computer screen, and to try to minimize blinks. The E-Prime software package (Psychological Software Tools) controlled stimulus presentation and behavioral data collection.

The participants completed half of the trials for each of the two EEG tasks during each visit. The order of completion of each set of trials for each task was counterbalanced across the visits as was the order of completion of each of the tasks at each visit. At the beginning of each session, prior to beginning the experimental tasks, all participants completed a series of practice trials for each task and no participants were excluded for failure to understand the tasks.

#### Semantic congruency task

The spoken version of the Holcomb et al. [34] semantic judgement task was used to assess adolescents’ lexical-semantic knowledge. The task was modeled after Kutas and Hillyard [48] but modified to contain topics and vocabulary suitable for children in the second grade. Adolescents listened to audio recordings of a total of 160 simple, declarative sentences, ranging in length from 3 to 13 words with topics and vocabulary suitable for second graders. Eighty of the sentences had a final word that was semantically congruent with the constraints of the preceding sentence context (cloze probability > .80) (e.g., Giraffes have long NECKS) and 80 sentences that ended in a word that was semantically incongruent with the preceding sentence context (e.g. Giraffes have long SCISSORS). The sentences were designed such that each target word served as both a congruent and incongruent ending, thus presenting confounding effects based on characteristics of the target word itself. The same presentation format as Holcomb et al., [34] was used where participants pressed the “yes” button if the sentence “made sense” and the “no” button if it did not.

The sentences were divided into four lists. Lists 1 and 3 contained the same sentence stems (the first 80 sentences) and lists 2 and 4 contained the remaining 80 sentences. In each list, 40 of the sentences ended with a congruent word and the other 40 sentences ended with an incongruent word. In list 3, the 40 congruent sentences from list 1 were repaired with final words that were incongruent, but still came from the same list, while the incongruent sentences were repaired so that they were fitted with their appropriate congruent ending. Lists 2 and 4 were created in the same manner from the remaining 80 sentences. Sentences were separated into four blocks of 20 trials each with short breaks between experimental blocks. Each participant was presented with two of the four lists (either lists 1 and 2 or lists 3 and 4) at the first visit and the remaining two lists were presented at the second visit. Each of the 80-item sets was presented in a fixed pseudo-random order, and the order of completion of the 80-item sets during the visits was counterbalanced across the groups. Prior to the task, participants completed ten practice trials. Each target word served both as a congruent and incongruent sentence ending.

The stimuli were the original recordings from Holcomb et al. [34]. Each sentence was spoken by a female experimenter at a slow speaking rate (one word per second). Because the original stimuli were originally recorded on analog tape, the files were digitized (16 kHz, 12-bit resolution). Stimuli were presented via E-Prime via ear inserts at a comfortable listening level (60-dB SL) with a stimulus-onset asynchrony (SOA) between words of 1000 ms. After successful completion of the practice trials, the experimental trials were presented. No participant was excluded for failure to understand the task, and no participant was excluded because of excessive artifacts in their EEG data.

#### Lexical decision task

Adolescents’ ability to retrieve words from their lexicon based on sensitivity to word frequency was assessed using a lexical decision task. Adolescents listened to a series of words and nonwords and were instructed to press the “yes” button if they thought what they heard was a real word, and the “no” button if they thought it was not a real word. To provide a sensitive measure of potential deficits in procedural memory, the words differed in word frequency—based on the PDH prediction that the ability to retrieve low-frequency (LF) words from one’s lexicons would be more vulnerable to procedural sequential deficits as compared to high-frequency (HF) words. Participants completed a total of 300 trials, one list of 150 of the trials (50 HF, 50 LF, 50 nonword) during the first visit and the remaining list of 150 during the second visit. Each of the lists was presented in a fixed pseudo-random order, and the order of completion of the lists during visits 1 and 2 was counterbalanced both within and across the two groups.

Stimuli consisted of a total of 300 words/nonwords, 100 HF words, 100 LF one-syllable CVC/CVCC words (nouns, verbs, and adjectives), and 100 pronounceable nonwords (Table 2). The HF and LF words differed significantly in word frequency *F* (1, 198) = 107.88, *p* < .0001, with the HF words having frequency ratings ≥ 40 per million and the LF words having frequencies ≤ 10 per million based on the MRC Psycholinguistic Database ([50], http://www.psy.uwa.edu.au/mrcdatabase/ uwa_mrc.htm). Both the HF and LF word lists had the same distribution of initial consonants [51]. All words had neighborhood density values of 4 o higher (Washington University in St. Louis Speech & Hearing Lab Neighborhood Database http://128.252.27.56/Neighborhood/Home.asp) and the HF and LF lists did not differ from each other in neighborhood density *F* (1, 198) = 0.00, *p* = .99. Imageability ratings of the words were set at 2.2 or higher based on the Psychonomic Society Web Archive (http://www.psychonomic.org/ ARCHIVE/) and Cortese & Fugett [52] and the HF and LF words did not differ significantly from each other in imageability *F* (1, 198) = 0.45, *p* = .51. Finally, initial consonants were controlled for the HF and LF word sets with both lists having the same distribution of consonants. The nonword foils were created from the HF and LF word lists by changing the final phoneme of the word to ensure that participants were not able to determine if the stimulus was a “word” or a nonword based on the initial CV or CCV portion of the acoustic signal. Half of the nonwords were created from HF words and half from LF words. All of the nonwords had the same distribution of initial CV combination as the HF and LF words.

**Table 2:** Lexical characteristics and comparisons of HF and LF words

		List^a^		*p*
		High	Low	High vs. low
Word frequency^b^	*M (SD)*	210.33 (200.18)	2.40 (1.99)	*p* < 0.001
	Range	40–1207	1–9	
Imageability^c^	*M (SD)*	5.06 (1.11)	5.15 (0.96)	*p =* 0.51
	Range	2–7	2–7	
Neighborhood density^d^	*M (SD)*	21.73 (6.56)	21.72 (6.22)	*p =* 0.99
	Range	4–36	9–35	

^a^*n* = 100 for each list

^b^MRC Psycholinguistic Database, http://www.psy.uwa.edu.au/mrcdatabase/uwa_mrc.htm, based on [49]

^c^MRC Psycholinguistic Database, http://www.psy.uwa.edu.au/mrcdatabase/uwa_mrc.htm [50]

^d^Washington University in St. Louis Speech & Hearing Lab Neighborhood Database, http://128.252.27.56/Neighborhood/Home.asp

The stimuli were digitally recorded in a sound-attenuating chamber, at 44.1 kHz. To eliminate audible “clicks” at the onset of each word, the first 10 ms of the acoustic waveform for each word was enveloped. To control potential dialectic effects on lexical processing, because the participants were all from California, the sound stimuli were recorded by a female speaker with a mid-central California dialect. Stimuli averaged 573.18 ms in duration (373–829 ms). For each trial, an ISI duration was randomly generated by E-Prime, seeded from a random numbers table, and constrained to only have values between 1000 and 2000 ms. To enable E-Prime to randomly generate the different ISIs a period of silence was added to the end of each sound stimuli so that every sound file was 1000 ms in total duration.

#### EEG data collection and processing

EEG data for both tasks were acquired with the Electrical Geodesics Inc. (EGI) high-density array 128 channel system consisting of Hydrocele Electrical Geodesic Ag/AgCl Sensor electrodes, Net Amps, and Net Station software (v 4.3.1 Electrical Geodesics Inc., Eugene OR) running on a Macintosh G4 computer. The net has pedestals marked with the vertex, ears, and nasion locations. The net’s anatomically marked locations were used to position the sensor net and the elasticity of the connections between pedestals positioned the other electrodes in a fixed set of positions on the scalp. The electrode positions cover the scalp with interelectrode distances of 35 to 40 mm. EEG data were sampled at 250 Hz (4-ms samples) with band-pass filters at 0.1–100 Hz, with 20K amplification during the experiments with Electrical Geodesics, NetAmps 200 amplifiers. Impedances were kept under 100 kΩ and did not differ significantly between the two groups (SLI: *M* = 42.62 kΩ, CA: *M* = 38.44 kΩ; *F* (1, 26) = 1.3, *p* = .26).

EEG signals were recorded with a vertex reference (Cz). After data collection was completed, EEG files were digitally low pass filtered (30 Hz) offline. Trials containing eye blinks (> 140μV), eye movements (> 55μV), or other artifacts (> 200μV) were marked using the Artifact Detection tool within the NetStation software package. Bad channels were removed from recordings and replaced with spherical spline interpolation from the remaining channels. Blink correction using the Ocular Artifact removal (OAR) NetStation Waveform Tool was then run on all participants’ EEG data. Artifact detection was run a second time to re-mark the Blink-corrected trials as usable. EEG signals were then re-referenced offline to an average of right and left mastoid electrodes and baseline corrected to a 100 ms pre-stimulus interval.

The number of bad channels that were removed for the semantic congruency task was low for both groups (SLI *M* = 8/128; CA *M* = 4/128) and did not differ between groups *F* (1, 26) = .81, *p* = .38. The total number of artifact-free trials for the semantic congruency task did not differ for the two groups for congruent sentences (SLI *M* = 59.5, *SD* = 16.5; CA *M* = 68.8, *SD* = 10.5) *F* (1, 26) = 3.164, *p* = .08 or for incongruent sentences (SLI *M* = 59.3, *SD* = 17.0, CA *M* = 69.4, *SD* = 8.1) *F* (1, 26) = 4.008, *p* = .07. The number of bad channels that were removed for the lexical decision task was also low for both groups (SLI *M* = 11/128; CA *M* = 9.4/128) and did not differ between groups *F* (1, 26) = .455, *p* = .55. The total number of correct, artifact-free trials for the lexical decision task differed by word frequency *F* (1, 26) = 70.902, *p* = .000, and group *F* (1, 26) = 4.53, *p* = .043 with more artifact-free trials for HF as compared to LF for both groups and more correct and artifact-free trials for the CA group as compared with the SLI group *F* (1, 26) = 4.44, *p* = .044 (HF: SLI *M* = 64.8, *SD* = 24.6; CA *M* = 78.7, *SD* = 11.5; LF: SLI *M* = 52.6, *SD* = 23.5, CA *M* = 68.6, *SD* = 12.7)

**Fig. 1:**
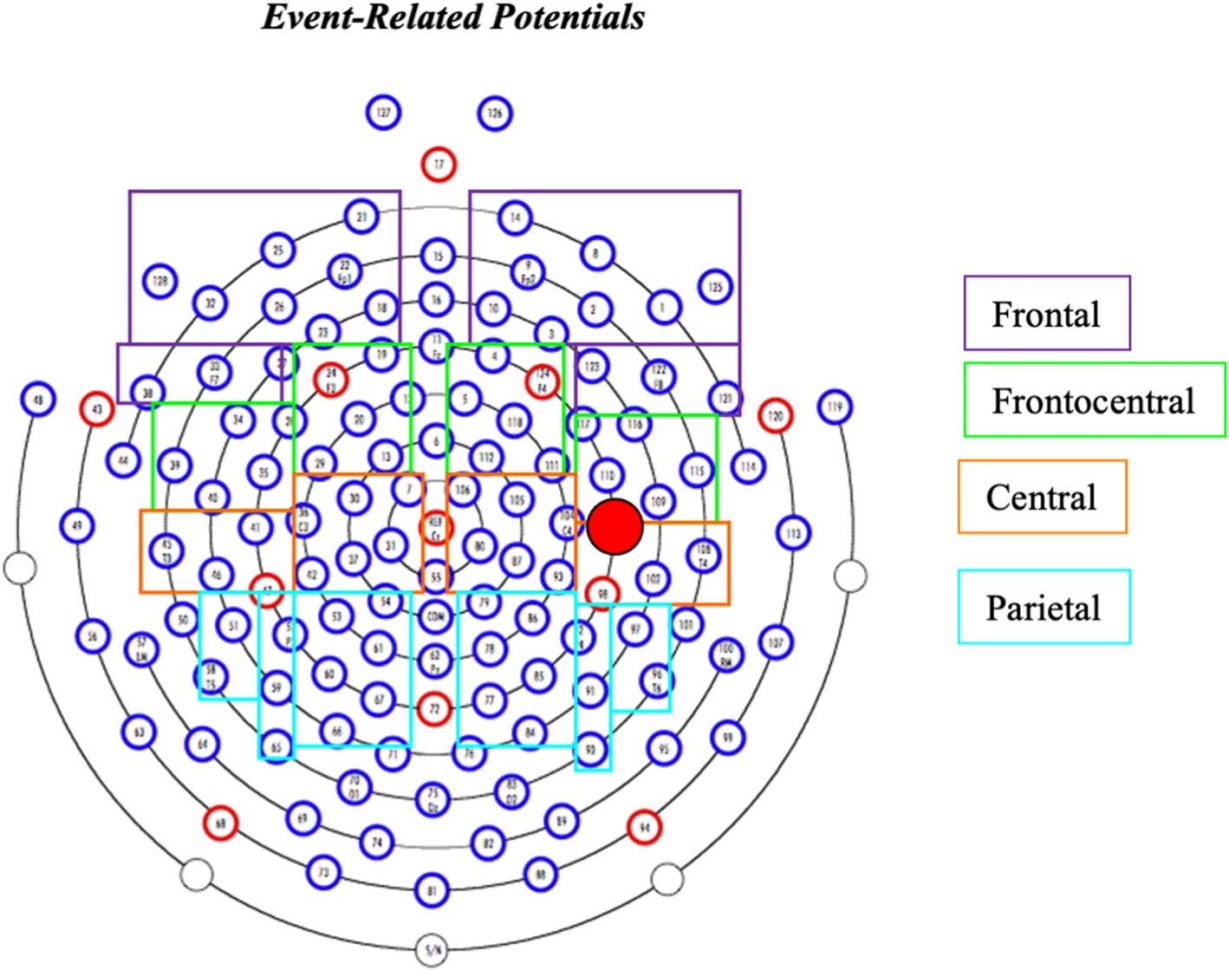
Channels for frontal, frontocentral, central, and parietal regions of interest (ROIs)

For the EEG analysis, epochs of 1300 ms, − 100 to 1200 ms relative to the target were created from the continuously recorded EEG data. For the semantic congruency and lexical frequency tasks, the temporal window for measuring ERP mean amplitudes was selected after the grand averages were examined and the windows were centered around the regions of maximal activity in each task [30]. Once identified for each task, the same temporal window was used for the SLI and CA group’s grand averages. Because the spatial distribution of the N400 differs somewhat by context for semantic congruency, word frequency, concreteness, etc., to capture potential differences in the distribution of the cortical response of the SLI and CA groups, we took advantage of the high-density recording and examined eight regions of interest based on an average of a combination of electrodes in left frontal, right frontal, left frontocentral, right frontocentral, left central, right central, left parietal, and right parietal regions (Fig. 1).

#### EEG analysis procedures

The ERP data were analyzed using a series of repeated measures ANOVAs. The omnibus models included laterality (left, right) and group (SLI, CA) for each of the ROIs with mean amplitude for either semantic congruency/incongruency or high/low word frequency as the dependent variables in each analysis. EEG data were then analyzed separately for each of the two groups to characterize the typical pattern of cortical activity as exhibited by the CA group for each task over each of the ROIs and then to ascertain if the pattern of cortical activity for the adolescents with SLI mirrored that of CA controls. To identify potential qualitative differences in the electrophysiological response to the lexical-semantic and lexical-phonological tasks, the grand average animated topographic maps and the Student’s *t*-test (*α* = .05) difference maps also were examined for the CA controls and adolescents with SLI. Lastly, the relationship between procedural memory (statistical learning) and the degree of modulation of the N400 by congruency/incongruency and high/low word frequency over the individual ROIs for each of the two groups was examined.

## Results

All behavioral and psychophysiological analyses were corrected using the Greenhouse-Geisser correction applied to probability values to adjust for repeated measures. In studies with small sample sizes, one concern is the lack of power to detect significant group effects. The critical theoretical question in this study is the potential atypical cortical response on the part of the adolescents with a documented history of SLI-based interaction between group and semantic congruency/lexical frequency. To examine this question, we leveraged the strength of the PCND project where the participants’ original diagnostic classification was known, however this resulted in only 28 participants (SLI =14; CA = 14). Because a sample size of at least 34 per group is necessary to detect a moderate interaction effect (*d* = .40), with alpha set at .05 and power set at .90 [53], we first characterized the normal pattern of activation based on the CA groups’ cortical response and then determined whether the same pattern was evident in the adolescents with SLI. We then conducted follow-up planned direct group comparisons to examine the degree of modulation of the N400 across the two groups.

### Statistical learning and declarative memory

**Fig. 2:**
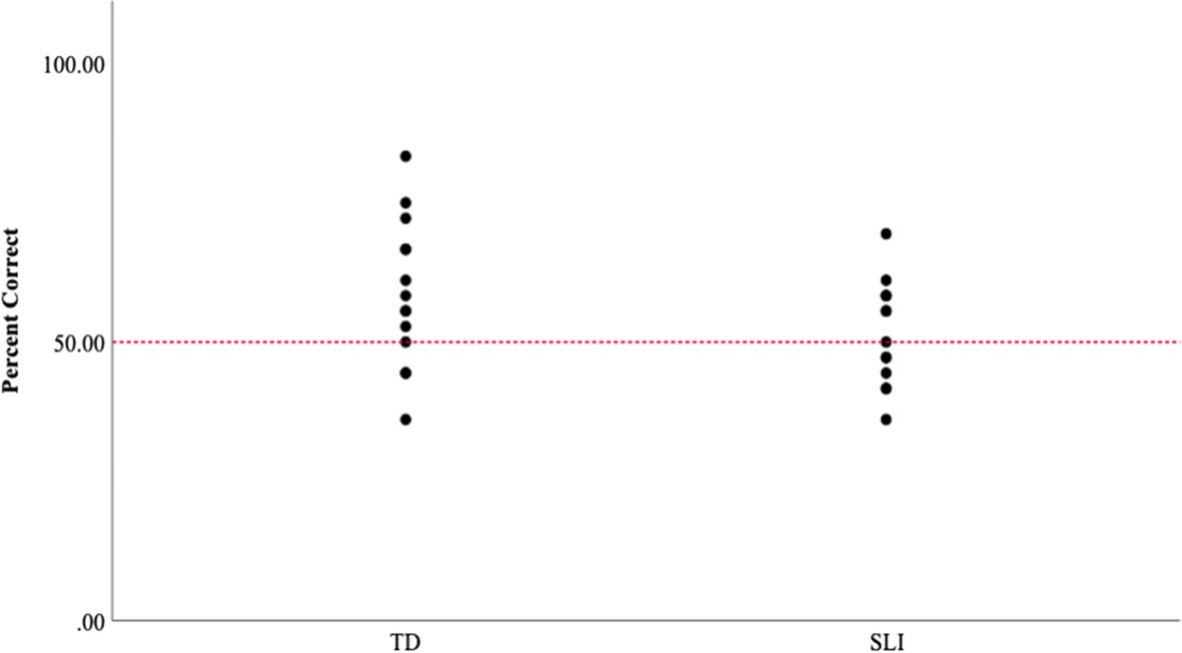
Comparison of performance for the groups for statistical learning

For the statistical word learning task, performance for the adolescents with SLI was no different from chance *t*(13) = .72, *p* = .48 whereas the performance for the CA group was significantly greater than chance *t*(13) = 2.49, *p* = .03 (SLI: *M* = 51%; *SD* = 9%; CA: *M* = 58%; *SD* = 13%); however, as a group, the performance of the adolescents with SLI did not differ from that of CA controls *F* (1, 26) = 2.62, p = .11. Accuracy on the Yes/No portion of the Competing Language Processing Task was high for both groups (SLI: *M* = 99%; *SD* = 1%; CA: *M* = 99%; *SD* = 2%) and did not differ for the SLI and CA controls *F* (1, 26) = .13, *p* = .72. Consistent with prior work, despite the SLI group’s ability to accurately evaluate the semantic content of the sentences on the Yes/No portion of the task, their ability to recall the last words of the sentences (i.e., verbal working memory) was significantly poorer than that of the CA controls *F* (1, 26) = 16.57, *p* < .00 (Fig. 2).

### Semantic congruency

**Fig. 3:**
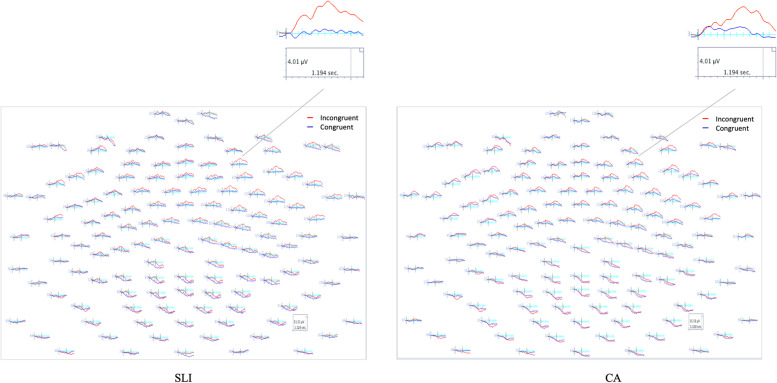
Head maps by group for semantically congruent and incongruent conditions

Behaviorally, the PDH predicts that the brain circuitry of the declarative memory system is largely intact in children with SLI. As such, those aspects of language hypothesized to be supported by the declarative system also should be intact in children with SLI (e.g., the encoding of episodic information, personal experiences and the arbitrary, idiosyncratic aspects of word knowledge and “semantics”). For the semantic congruency task, accuracy was high for both groups (SLI *M* = 95.61, *SD* = .041; CA *M* = 97.92, *SD* = .016) and did not differ *F* (1, 26) = 3.97, *p* = .06.^1^ Grand average waveforms for the congruent and incongruent conditions for the SLI and CA groups time-locked to the onset of the word are shown in Fig. 3. For the CA group, in the frontocentral channels, beginning approximately 300 ms after the word onset and lasting through the end of the epoch, the most notable pattern is the large negative deflection (N400). The second most notable pattern is the greater negative deflection of incongruent as compared to congruent endings during the 500–800 ms time window.

For the CA group, a repeated measures ANOVA including condition (congruent, incongruent), laterality (left, right), and region (frontal, frontocentral, central, parietal) revealed a significant main effect of congruency *F* (1,13) = 4.83, *p <* .05 and region *F (*1,13) = 98.26, *p <* .00, and significant congruency by laterality *F* (1,13) = 11.59, *p <* .00, and congruency by region *F* (1,13) = 6.59, *p <* .00 interactions. Post hoc analysis revealed that for the CA group the mean amplitude for the incongruent condition was significantly more negative as compared to the congruent condition over the right frontal regions *F* (1,13) = 8.57, *p <* .01, bilaterally over both the right frontocentral regions *F* (1,13) = 11.39, *p <* .01 and left frontocentral regions *F* (1,13) = 5.00, *p <* .05, and over the right central regions *F* (1,13) = 9.25, *p <* .01. For the SLI group, a repeated measures ANOVA revealed a significant main effect of congruency *F* (1,13) = 9.12, *p <* .05 and region *F* (1,13) = 22.57, *p <* .00, and significant congruency by laterality *F* (1,13) = 12.24, *p <* .00, but no congruency by region *F* (1,13) = .64, *p =* .43 interaction. Post hoc analysis revealed that for the SLI group, the mean amplitude for the incongruent condition was significantly more negative as compared to the congruent condition over the right frontal region *F* (1,13) = 13.39, *p <* .01, right frontocentral region *F* (1,13) = 11.39, *p <* .01, and bilaterally over both the right central *F* (1,13) = 15.99, *p <* .01, and left central region *F* (1,13) = 8.24, *p <* .05. A repeated measures ANOVA including group, laterality, and region indicated that the mean amplitude for congruent word endings was the same for the SLI and CA groups over the right frontal *F* (1, 26) = .56, *p =* .45; frontocentral *F* (1, 26) = .44, *p =* .51; central *F* (1, 26) = .15 *p =* .69; or parietal regions *F* (1, 26) = .46, *p =* .50. Similarly, the mean amplitude of response to incongruent endings was the same for the SLI and CA groups over the right frontal *F* (1, 26) = .17, *p =* .67; frontocentral *F* (1, 26) = .06, *p =* .80; central *F* (1, 26) *=* .00 *p =* .92; or parietal regions *F* (1, 26) = 1.6, *p =* .27.

#### Animated topographies

To examine changes in scalp topography over time for the SLI and CA groups, the data were interpolated onto two-dimensional scalp topographies over the event windows in question. In contrast to the static grand average waveforms, these topographies make it easier to examine temporal and spatial aspects of the data for the two groups. In addition, Student’s *t*-tests—means normalized by their standard errors—topographic maps (*α* = .05) also were calculated for each group to examine potential differences between processing of semantically congruent and incongruent words, normalized by the variance across the participants within each group. Although these *t*-animations were post hoc, because the difference waves permit visual “decomposition” of the waveform taking into account temporal covariance, they are valuable in examining potential differences in the two groups regarding the number of potential neural sources that could be generating the effects as well as their interactions [54].

**Fig. 4:**
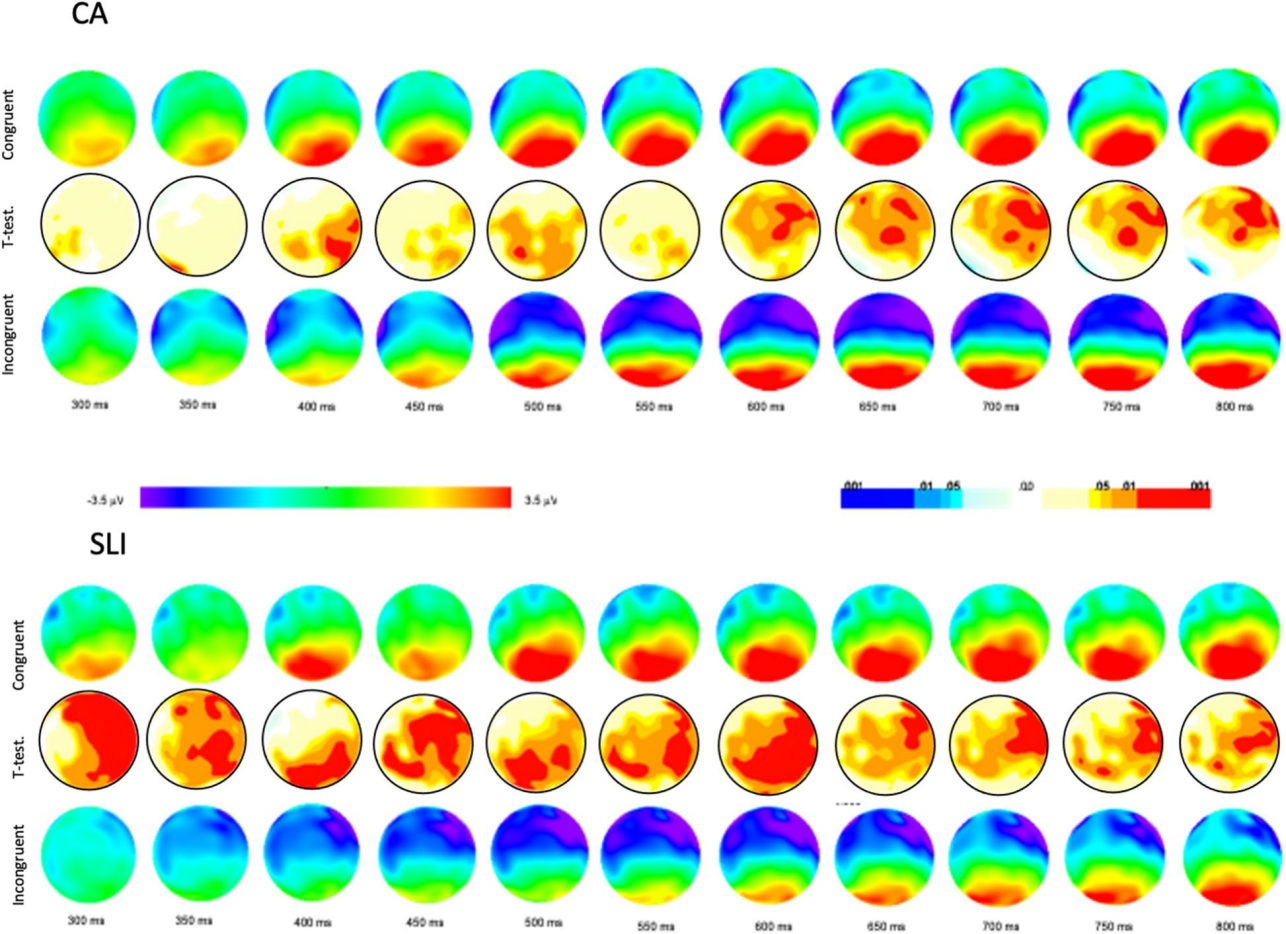
Topography maps by group for semantically congruent and incongruent conditions

The grand average topographic maps and the Student’s *t*-test (*α* = .05) difference maps for the congruent and noncongruent word conditions—top and bottom maps—for the two groups are shown in Fig. 4. For the CA controls, for the incongruent condition, beginning at approximately 300 ms an increased negative was evident bilaterally and then becoming more negative over anterior and central sites beginning at approximately 550 ms after the onset of the target word and continuing over the course of the epoch. For the adolescents with SLI, for the incongruent condition, beginning at approximately 350 ms after the onset of the target word, increased negativity was evident over anterior and central sites, becoming somewhat diminished in negativity beginning approximately 600 ms and continuing to diminish over the course of the epoch.

The difference maps—middle plot between the maps for the congruent and incongruent conditions—show regions where the amplitude of the congruent and incongruent conditions differed statistically significantly (*t*-test *α* = .05) over the time course of the epoch. For the CA controls, there was a statistical difference in the negativity of the incongruent as compared to congruent word endings in parietal regions that was right lateralized to incongruent relative to congruent word endings (center maps) occurring briefly at approximately ~ 400 ms, and then again at ~ 500 ms. Then, beginning at approximately 600 ms after the onset of the target word, response to incongruent word endings shifted to being significantly different from congruent word endings over right-lateralized central and parietal regions continuing over the course of the epoch.

The pattern differed for the SLI group. A largely right-lateralized increase in negativity in response to incongruent word endings was evident from 300 to 400 ms. This response to incongruent word endings then shifted briefly to a bilateral and posterior response at ~ 400 ms, and then shifted back to broadly right-lateralized increase in negativity from ~ 450 to 600 ms. Beginning at ~ 650 ms after the onset of the target word, the increase in negativity in response to incongruent word ending shifted to right-lateralized central and parietal regions and continuing over the course of the epoch. The groups were similar in that the response was characterized by an increase in negativity to incongruent word endings as opposed to a decrease in negativity to congruent word endings. Differences in the time course and location of this increased negativity to incongruent word endings also was evident for the two CA and SLI groups, however, indicating overlapping and non-overlapping neural sources for the two groups.

#### Semantic congruency summary

Similarities and differences were observed in the cortical response to semantic congruency for the two groups. Between 500 and 800 ms after the onset of the target word, for the CA controls the response to semantic congruency was characterized by the mean amplitude for the incongruent condition being significantly more negative than the congruent condition bilaterally over the frontocentral ROIs and in the right hemisphere over the frontal, central and parietal regions. For the adolescents with SLI, the response to semantic congruency differed slightly from that of the CA controls with the mean amplitude for the incongruent condition being significantly more negative in the right hemisphere over the frontal and frontocentral regions and then bilaterally more posterior over the central regions. This difference in the pattern of cortical response in response to semantic congruency was evident in the Student’s *t*-test (*α* = .05) difference maps as well. The pattern for the CA controls was distributed predominantly over the right hemisphere whereas the adolescents with SLI exhibited a more bilaterally distributed pattern of activation.

### Lexical decision

The PDH predicts that procedural memory deficits should be linked to impaired morphosyntax and grammar in children with SLI. The PDH also predicts that impaired procedural memory will be linked to slower less accurate lexical retrieval and reduced sensitivity to word frequency as evidenced by slower, less accurate processing of low- as compared to high-frequency words. Although both groups were more accurate in detecting high-frequency words from nonwords (SLI *M* = 87%; CA *M* = 95%) as compared to detecting low-frequency words from nonwords (SLI *M* = 67%; CA *M* = 82%) *F* (1, 26) = 140.495, *p <* .00, overall, accuracy was poorer for the SLI group as compared to the CA controls in detecting both HF and LF words from nonwords *F* (1, 26) 10.448, *p <* .01. *A* also was calculated for each participant.^2^ Nonparametric statistical analysis confirms the above findings, where detection of words from nonwords was significantly higher for both groups for HF as compared to LF words (SLI: Wilcox signed ranks test *z* = − 3.29, asymptote sig. 2-tailed = .001; CA: Wilcox signed ranks test *z* = − 3.29, asymptote sig. 2-tailed = .001), however, detection of words versus nonwords was significantly lower for the adolescents with SLI as compared to CA controls for both HF (Mann-Whitney *U* = 40, asymptote Sig 2-tailed = .008) and LF words (Mann-Whitney *U* = 27.4, asymptote sig 2-tailed = .001).

Reaction times were measured from the onset of the stimulus item until a button press was recorded. Only correct trials were included in the RT analysis. Both groups’ reaction times were significantly faster for the HF (SLI *M* = 1125.94 ms, range 1005.06–1316.22 ms; CA *M* = 1109.26 ms, Range 887.26–1290.07 ms) as compared to LF frequency words (SLI *M* = 1200.71 ms, Range 1075.39–1414.40 ms; CA *M* = 1178.14 ms, Range 1002.72–1366.25 ms) *F* (1, 26) = 135.93, *p* < .0001. Although the adolescents with SLI were less accurate in determining word versus nonword status as compared to CA controls, they were no slower in detecting both HF and LF frequency words from nonwords *F* (1, 26) = .002, *p* = .97.

**Fig. 5:**
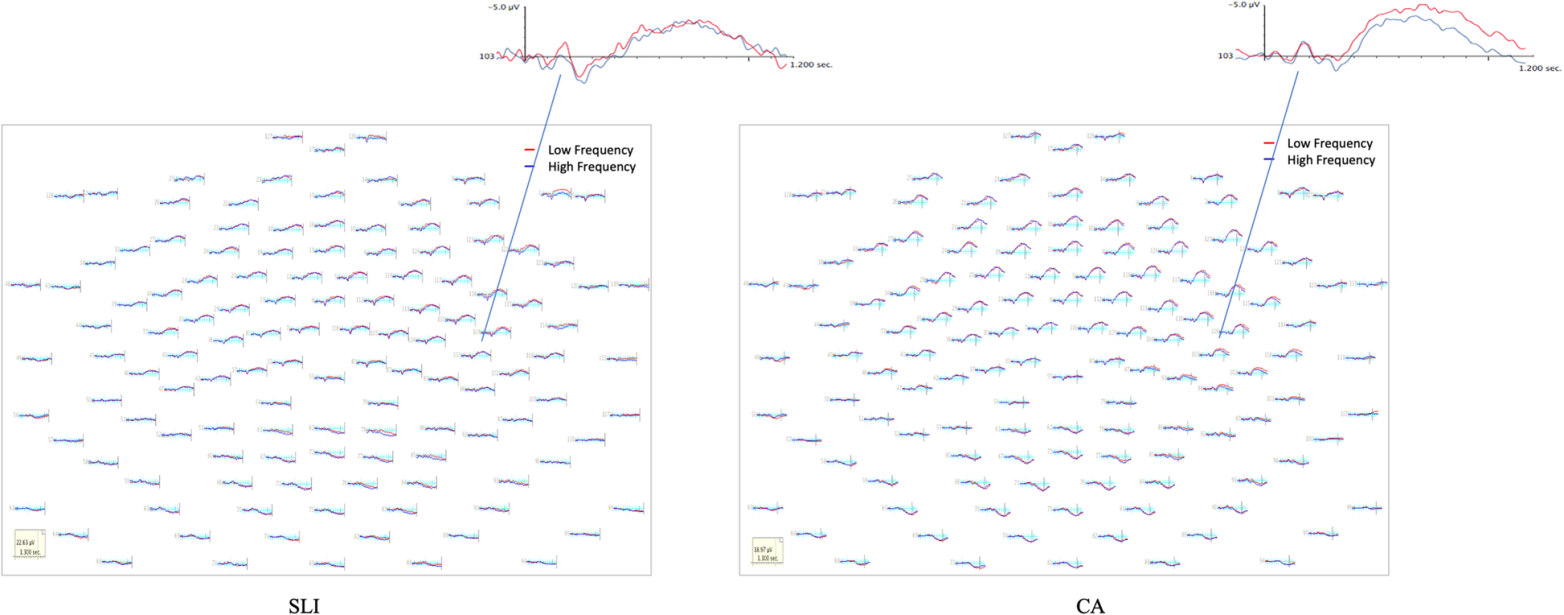
Head maps by group for high and low word frequency conditions

Grand average waveforms for the HF and LF word conditions for the SLI and CA groups time-locked to the onset of the word are shown in Fig. 5. Beginning approximately 500 ms after the word onset and lasting through the end of the epoch, the most notable pattern is the large negative deflection (N400). The second most notable pattern is the greater negative deflection of LF as compared to HF words during the 700–1200-ms time window. Although the overall pattern was similar for the adolescents with SLI and CA controls, visually, there is one major difference in the waveforms of the N400 for the two groups—notably there was no negative deflection of LF as compared to HF words for adolescents with SLI.

For the CA group a repeated measures ANOVA including word frequency (High, Low), laterality (Left, Right) and region (Frontal, Frontocentral, Central, Parietal), revealed significant frequency by laterality *F* (1, 13) = 5.58, *p* < .05 and frequency by region *F* (1, 13) = 6.03, *p* < .05 interactions. Post hoc analysis reveals that for the CA group, the mean amplitude for the LF words was significantly more negative than HF words over the right central region *F* (1, 13) = 6.78, *p* < .02. For the SLI group a repeated measures ANOVA revealed no effect of frequency *F* (1, 13) = .39, *p* =.53, frequency by *laterality F* (1, 13) = 2.78, *p* = .11, or frequency by region *F* (1, 13) = .15, *p* = .70 interactions. Direct comparison of the mean amplitude for HF words over the right central region revealed no difference for the CA and SLI groups *F* (1, 26) = .19, *p* = .66 (SLI *M* = − .98 CA *M* = − 1.34); however, comparison of the mean amplitude for LF words over the right central region was not significance *F* (1, 26) = 3.15, *p* = .08, (SLI *M* = − 1.2 CA *M* = − 2.7) suggesting that the lack of modulation by word frequency for the SLI group was due to a less robust response to LF words as compared to nonwords as opposed to HF words compared to nonwords.

#### Animated topographies

**Fig. 6:**
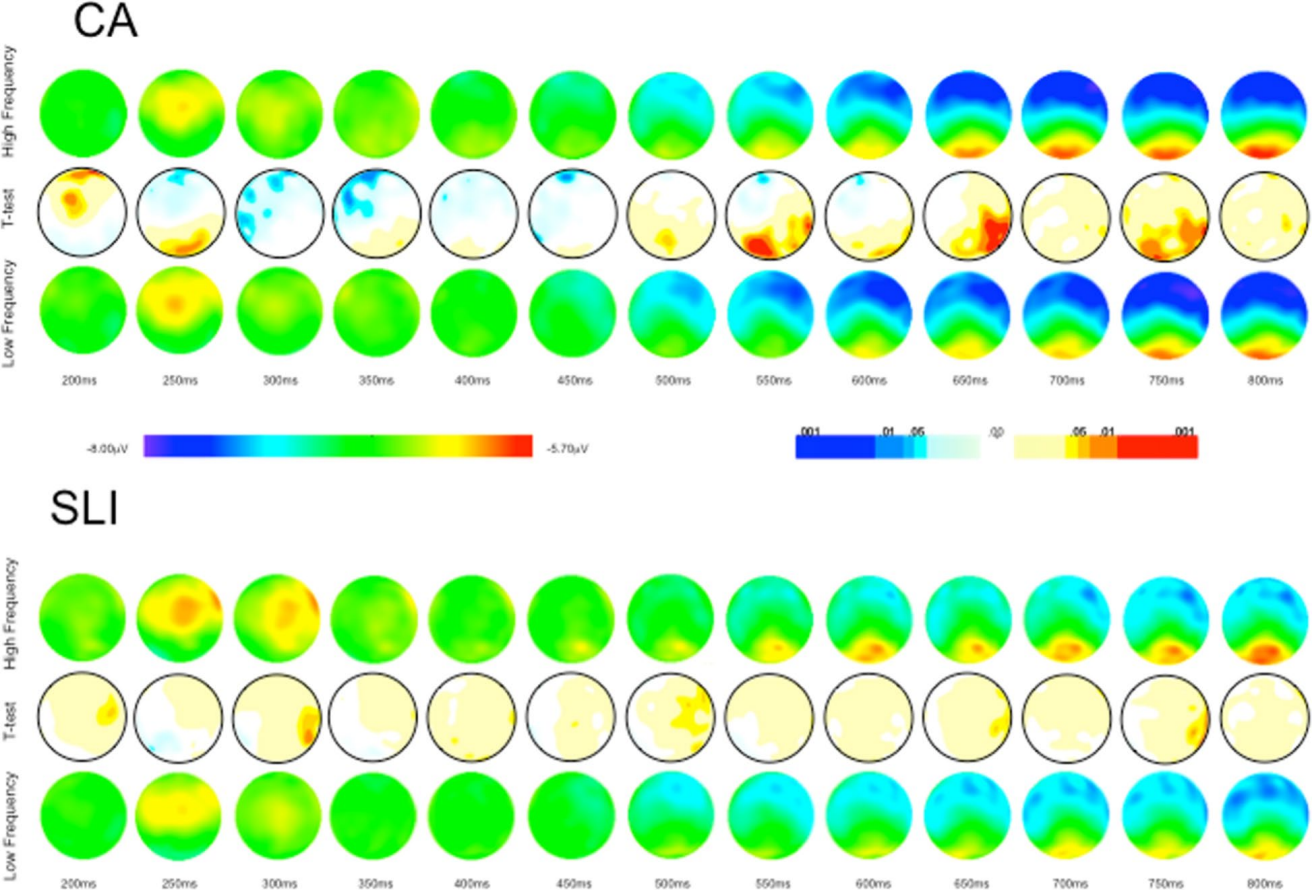
Topography maps by group for high and low word frequency conditions

The topographic maps and Student’s t-test difference maps (*α* = .05) between the HF and LF word conditions for the SLI and CA controls are shown in Fig. 6. For the CA group, for low-frequency words, increased negativity is evident in bilateral anterior sites beginning approximately 500 ms after the onset of the target word and continuing to the end of the epoch. The difference map—middle plot—for the high- and low-frequency words for the CA group showed a statistical difference in amplitude for LF as compared to HF words, characterized by an increase in negativity to LF words over right centro-parietal sites beginning approximately 600 ms after the onset of the target word. In contrast, as can be seen in Fig. 6, there was no evidence of increased negativity to low-frequency words for the adolescents with SLI and the difference map revealed no statistically significant difference in the amplitude of high and low-frequency words for the adolescents with SLI.

#### Procedural memory and lexical frequency

The PDH predicts that procedural memory ability should be linked to lexical-phonological processing, in particular speed of processing and sensitivity to word frequency. For the CA controls, statistical word learning ability was not significantly correlated with accuracy of detecting words from nonwords for either HF *r*(14) = .34, *p* = .22 or LF words *r*(14) = .46, *p* = .09, but was negatively correlated with CA participant’s speed of detecting words from nonwords (RT) for HF words *r*(14) = − .53, *p* < .05 but not for LF words *r*(14) = − .52, *p* = .06. This indicates that for the CA controls, those participants who were better able to implicitly track the transitional probability of phonemes within the stream of speech also were faster to identify HF words from nonwords. Although statistical word learning ability was related to the speed of processing for the CA controls, it was not significantly correlated with the degree of modulation of the N400 by HF versus LF words over left or right frontal, frontocentral, central, or parietal ROIs for the CA controls. For the adolescents with SLI, statistical word learning ability was not significantly correlated with accuracy in detecting words from nonwords for either HF *r*(14) = − .13, *p* = .63 or LF words *r*(14) = .08, *p* = .78, or in their speed (RT) of detecting words from nonword for HF *r*(14) = .43, *p* = .12 or LF words *r*(14) = .39, *p* = .16. Similarly, for adolescents with SLI, statistical word learning ability was not correlated with modulation of the N400 by word frequency over left or right frontal, frontocentral, central, or parietal ROIs.

Follow up analysis of the correlation coefficients for statistical word learning and accuracy for high- and low-frequency words for the CA and SLI groups revealed that the correlations for the two groups did not differ for accuracy for HF *z* = − .24, *p* = .40 or LF words *z* = .97, *p* = .16, but the correlation coefficients for statistical word learning and RT was statistical significantly different for the CA and SLI groups for both HF words *z* = 2.46, *p* < .001 and LF words *z* = 2.31, *p* < .05 indicating that relationship between statistical word learning and speed of lexical processing for both high- and low-frequency words was significantly different for the CA controls as compared to the adolescents with SLI

#### Lexical decision summary

For the CA controls, beginning ~ 700 ms after the onset of the target word, the response to lexical-phonological information in the form of word frequency was characterized by the mean amplitude for LF words being significantly more negative than HF words in the right hemisphere over the frontocentral and central ROIs. The topographic maps and Student’s *t*-test difference maps show the modulation of the cortical response by word frequency in the right hemisphere over frontocentral and central regions. For the CA controls, procedural memory was correlated with the degree of modulation of the cortical response to word frequency over the right central ROI accounted for a small unique amount of variance in the cortical response to word frequency over this region as well.

In contrast, modulation of the cortical response by word frequency was absent for the adolescents with SLI with the topographic maps and Student’s *t*-test difference maps highlighting the notable absence in the modulation of the cortical response by word frequency for the adolescents with SLI. Similarly, no correlation was evident in SWL performance or modulation of the cortical activity in response to word frequency over any of the ROIs, and regression analysis revealed that SWL performance did not account for any variance in the modulation of the cortical response to word frequency for the adolescents with SLI.

### Lexical decision: Imageability

The PDH not only predicts poor sensitivity to word frequency on the part of adolescents with SLI but also predicts the compensatory use of declarative memory processing strategies by these individuals. Importantly, however, Ullman and colleagues have suggested that compensatory reliance on declarative memory may by individuals with SLI may only be evident when the neural underpinnings of language processing also are examined [5, 6].. Given the behavioral data showing that the adolescents with SLI were able to detect words from nonwords, the lack of modulation of the N400 by word frequency for this group was unanticipated and suggests that they may have been using a different cognitive strategy to determine if the stimulus was a “word” or a “nonword” as compared to the CA controls. With respect to language, the declarative memory system not only supports the acquisition of semantic and episodic knowledge in the traditional sense of “meaning”, but also words (i.e., lexical knowledge), images, and event knowledge [57, 58]. Dual-coding theory suggests that the mental lexicon includes both perceptual and symbolic semantic representations and that these two different aspects of the mental lexicon are supported by declarative and nondeclarative memory systems, respectively [59, 60] where lexical knowledge acquired via the declarative memory system has been posited to be concrete, grounded, and perceptually rich in nature [61]. Although Paivio [59] proposed that imageability and concreteness were merely alternative measures of mental imagery, EEG studies have shown that the effects of imageability and concreteness are distinct and dissociable and evident in both the early (500–700 ms), and late N700 (500-700 msec) time windows [34, 36]. For example, Bechtold and colleagues [36] observed no effect of imageability in either the N400 (300–500 ms) or the early N700 time window (500–700 msec). They did, however, observe an effect for imageability during the late N700 time window (700–900 ms), where highly imageable words consistently elicited significantly more negative amplitudes over frontal regions as compared to low imageable words.

Because this study examined the cortical response from – 100 to 1200 ms, it allowed us to look for evidence of an N700 effect, which would suggest the possible compensatory use of a strategy that relied on the imageability of the words—the ease with which the participants could form a mental image of a word based on episodic/conceptual knowledge of the words—on the part of the adolescents with SLI. In a post hoc analysis, using a median split, we regrouped the words from the lexical decision task based on the imageability rating of each of the words. The result was two new word lists that differed in imageability but which did not differ in word frequency (see Table 3). Importantly, word frequency and imageability ratings of the words were not correlated in the reorganization of the word lists based on imageability (*r* = − .092, *p* = .20).

**Table 3:** Lexical characteristics of high-imageability words (high) and low-imageability words (low)

		High	Low	High vs. Low
		*n* = 103	*n* = 97	
Word Frequency^a^	*M (SD)*	97.26 (170.94)	114.40 (180.55)	*p* = 0.49
	Range	1–1207	1–967	
Imageability^b^	*M (SD)*	5.93 (0.57)	4.24 (0.63)	*p* < 0.0001
	Range	5.1–6.9	2.2–5.0	

^a^MRC Psycholinguistic Database, http://www.psy.uwa.edu.au/mrcdatabase/uwa_mrc.htm, based on Kucera & Francis, 1967

^b^MRC Psycholinguistic Database, http://www.psy.uwa.edu.au/mrcdatabase/uwa_mrc.htm, Cortese & Fugett (2004)

Accuracy in detecting words versus nonwords based on word imageability was significantly poorer for the adolescents with SLI as compared to CA controls for both highly imageable words (SLI *M* = 80%; CA *M* = 89%) and low imageable words (SLI *M* = 76%; CA *M* = 87%) *F* (1, 26) = 10.358, *p* < .01. Similarly, *A* for adolescents with SLI was significantly lower than CA controls for both high (Mann-Whitney *U* = 37.5, asymptote sig 2-tailed = .005) and low imageable words (Mann-Whitney *U* = 24.5, asymptote sig 2 tailed = .001), and did not differ for adolescents with SLI for high- versus low-imageability words (Wilcox signed ranks test *z* = − 1.788, asymptote sig. 2-tailed = .07), nor for CA controls (Wilcox signed ranks test *z* = − 1.524, asymptote sig. 2-tailed = .13).

**Fig. 7:**
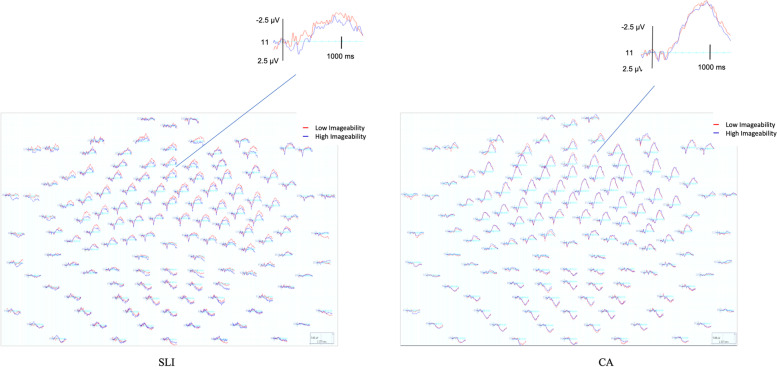
Head maps by group for high and low word imageability conditions

Grand average waveforms for the SLI and CA groups based on word imageability are shown in Fig. 7. Beginning approximately 300 ms after the word onset and lasting through the end of the epoch, the most notable pattern is the large negative deflection (N400) over the anterior regions. For the CA controls, a repeated measures ANOVA revealed that there was no effect on the mean amplitude of words based on imageability over frontal *F* (1, 13) = .006, *p* = .99, frontocentral *F* (1, 13) = .012, *p* = .91, central *F* (1, 13) = .077, *p* = .78 or parietal regions *F* (1, 13) = .121, *p* = .73. For the adolescents with SLI, word differing in imageability modulated cortical activity broadly and bilaterally over frontal regions *F* (1, 13) = 5.395, *p* < .05 and frontocentral ROI *F* (1, 13) = 4.42, *p* < .05, where words having low-imageability ratings elicited more negative amplitudes than high imageability words, but no modulation based on imageability was evident over the central ROI *F* (1, 13) = 1.524, *p* = .23 or parietal regions *F* (1, 13) = .014, *p* = .90.

## Summary of results and discussion

The purpose of this study was to leverage behavioral and EEG measures of lexical processing in adolescents with and without SLI to examine the PDH’s predictive relationship between declarative and procedural memory and lexical-phonological and lexical-semantic processing in a group of adolescents with a documented history of SLI. The PDH account predicts that declarative memory along with real-world lexical-semantic and conceptual knowledge should be intact in adolescents with SLI, whereas procedural memory should be impaired in these same individuals and linked to poor lexical processing. The PDH account also predicts that adolescents with SLI may rely on intact declarative memory as a compensatory language processing strategy but that this compensation may only be evident in the neural underpinnings of lexical processing as the behavioral performance of adolescents with SLI may overlap with that of CA controls and “appear” normal.

In the current study, procedural memory, as measured by statistical word learning, was greater than chance for CA controls but no different from chance for the adolescents with SLI, whereas real-world semantic knowledge as measured by the Yes/No questions of the Competing Language Processing Task was no different for the two groups. Behaviorally, accuracy on the semantic judgement task was high and did not differ for the two groups. The pattern of cortical activation on the semantic judgement task revealed both similarities and differences for the adolescents with SLI as compared to the CA controls. Although the grand average waveforms and topographic and difference maps in response to modulation of the cortical response by semantic congruency were broadly similar for both groups and characterized by increased negativity to incongruent responses as opposed to decreased negativity of the congruent responses, differences in the location and time course of the cortical response were evident as well. In particular, the topographic and difference maps for the two groups suggest that the cortical response to semantic congruency was driven both by overlapping neural sources common to both groups and possibly atypical, non-overlapping neural sources unique to the SLI group.

Behavioral accuracy for the lexical decision task suggested sensitivity to word frequency for both groups, although the adolescents with SLI were less accurate in their ability to determine word/nonword status for both HF and LF words as compared to CA controls. Both topographic and difference maps revealed a cortical response to spoken word word-frequency for the CA controls consistent with N400 frequency effects reported in adults [35, 62]. In contrast, there was *no* modulation of the cortical response by word frequency for the adolescents with SLI. To explore the possibility that the adolescents with SLI were using a compensatory strategy to determine word/nonword status on the lexical decision task, the data were reanalyzed based on word imageability. This analysis revealed a cortical response for the adolescents with SLI that was modulated by imageability in a bilateral manner over the frontal and frontocentral regions. In contrast, no modulation of the cortical response was evident based on word imageability over any ROIs for the CA controls.

Taken together, the findings from this study raise the possibility that, unlike the CA controls, the adolescents with SLI may have been relying on a real-world, “conceptual” lexical representations for both the semantic judgement and lexical decision tasks. Studies of sentence comprehension in SLI show that, well into the school-aged years, children with SLI are unable to process syntactic cues, relying instead on real-world knowledge of the physical properties of objects and events of the words to infer the meaning of sentences [63, 64]. The PDH predicts that lexical-semantic representations are intact in adolescents with SLI; however, a recent eye-tracking study with these *same* participants raises questions regarding the degree to which lexical-semantic representations are in fact intact. Specifically, Borovsky et al., [26] used eye tracking to examine lexical activation in the comprehension of simple transitive sentences in the participants as the current study. The adolescents listened to sentences of the form Article-Agent-Action-Article-Theme (e.g., *The pirate chases the ship*) while viewing pictures of four objects varying in the relationship to the Agent and Action of the sentence. Although the behavioral accuracy of the adolescents with SLI was no different from the CA controls, the adolescents with SLI evidenced a qualitatively different visual fixation pattern due to degraded underlying lexical-semantic representations.

In the current study, both groups were sensitive to word frequency effects in the lexical decision task. Although the CA group evidenced a cortical response to word frequency, the adolescents with SLI did not. In contrast, adolescents with SLI evidenced a cortical response to word imageability, suggesting that they were employing a qualitatively different strategy to determine “word” status as compared to CA controls. In particular, the modulation of the cortical response by imageability for the adolescents with SLI group suggests that they may have been forming a mental image of the words to determine word/nonword status, whereas the CA controls were determining word status based on the phonological form of the words/nonwords. Kounios & Holcomb [65] posit that the greater neural activity recruited to process highly imageable concrete words, as reflected by larger amplitude N400s, is consistent with the idea that concrete, highly imageable words activate more semantic information as compared to abstract, low imageable words. Concreteness and imageability often are viewed as the same construct in the literature; however, concreteness and imageability have been shown to represent two distinct, yet correlated, factors [66]. In the current study, all the words had high concreteness rating and did not differ across the high-/low-imageability lists. Therefore, we might not have expected N400 amplitudes for high-as compared with low-imageability words to pattern identically to concreteness effects, whereas N400 amplitudes have been reported to be more negative for concrete as compared with abstract words.

The findings from this study are consistent with those reported by Brown and Evans in an aMEG study with the same participants as the current study [32]. They asked if the dynamic functional brain organization for semantic processing of concrete objects differed for an adolescent with SLI as compared to that of CA controls. Using a “shoe box” task where participants were asked to determine if an object “fits in a shoe box”, they observed that on the word version of the task, behavioral accuracy for the adolescent with SLI overlapped with that of the normal controls, but that the adolescent with SLI showed no cortical activity within the left hemisphere. In contrast, he exhibited a pattern of atypical right hemisphere activity that was not evident in the controls. During evocation of the same semantic construct via homologous picture stimuli, the dynamic functional organization for this adolescent with SLI again was characterized by a pattern of under-recruitment of left hemisphere regions, but spatiotemporal activity that was strongly right lateralized. Based on these findings from the current study and that of Borovsky et al., consistent with Brown and Evans [33] proposal, there may be multiple pathways to language learning which may be evidenced by the atypical functional brain organization for adolescent with SLI.

In the current study, for CA controls statistical word learning ability was not significantly correlated with accuracy on the lexical decision task but was significantly correlated with speed of processing, where better statistical word learning ability was related to faster detection of HF words versus nonwords. In contrast, for the adolescents with SLI statistical word learning was not significantly correlated with either accuracy or speed of processing on the lexical decision task. With respect to the link between procedural memory and lexical processing hypothesized by the PDH account, statistical word learning was not significantly correlated with the modulation of the cortical response by word frequency for any ROI for either the adolescents with SLI or CA controls. The “at chance” statistical word learning performance by the adolescents with SLI may have obscured the detection of any relationship between procedural memory and lexical processing in this group, however, the absence of a link between procedural memory and cortical response for the CA controls suggests that the hypothesized relationship between procedural memory and language may not be as direct or transparent as posited by the PDH.

There has been considerable debate regarding whether procedural memory impairment is the underlying cause of language deficits in children with SLI [7–10, 67]. Procedural memory is a type of non-declarative/implicit memory [cf. 73,74,75]. In adult studies, the paradigmatic tasks used to examine procedural memory typically include motor learning (e.g., SRT), and artificial grammar learning tasks. Consistent with the findings of the first procedural learning study in SLI using this type of SRT task [68], poor procedural memory in SLI has consistently been reported in studies follow-up using SRT and artificial grammar tasks [7–10, 24]. For example, a meta-analysis by Obeid, et al. [9] examined a range of procedural learning tasks (i.e., SRT, Artificial Grammar Learning, Probabilistic Classification, etc.) and found that children with SLI consistently showed significant procedural learning impairment as compared to controls, with task modality (visual vs. auditory) not being a variable that moderated their observed effect sizes.

Non-declarative memory is not a single construct, however, but a term used to characterize a type of learning that occurs on an ongoing basis over multiple trials or exemplars and where learning is manifested in the gradual changes in performance or behavior across these tasks. In contrast to SRT or artificial grammar tasks, a paradigmatic measure of implicit learning in infancy and childhood is the ability to track the patterns of regularities across speech sounds that are contained within the speech stream to “discover” word boundaries. In these implicit learning studies, infants are exposed to a stream of elements that are constructed according to a specific set of regularities (i.e., statistics). The underlying assumption is that children and infants are implicitly tracking the statistical regularities in the speech stream. Results of these studies show that infants and children can effortlessly attend to and track a statistic known as transitional probability—the probability of occurrence between and across a sequence of syllables or phonemes—to learn which sounds co-occur with greater regularity than others. Moreover, they easily use this information to subsequently discover “word” boundaries within the stream of speech and readily use, and prefer, these newly extracted “words” as labels to novel objects [21].

Ullman and colleagues et al. [6] have argued that children also leverage procedural memory to implicitly track and segment sequences of syllables or phonemes from the speech stream in statistical word learning studies. Evans and colleagues [22] were the first to observe that statistical word learning was impaired in children with SLI. They also observed that for both typical children as well as children with SLI, statistical word learning ability was directly linked to both expressive and receptive vocabulary. Ahufinger and colleagues [23] recently examined statistical word learning in English and Catalan-Spanish speaking children with and without SLI. They also observed the same pattern as Evans and colleagues. Statistical word learning performance was impaired in their Catalan-Spanish speaking children, but not in their age- and IQ-matched normal language controls. Moreover, regression models revealed that for both the English and Catalan-Spanish speaking SLI and CA cohorts, statistical word learning was directly linked to vocabulary, accounting for a significant amount of unique variance in both receptive and expressive vocabulary in both language cohorts. Further, likelihood estimates revealed that for both the English speaking and Catalan-Spanish speaking cohorts, statistical word learning ability diagnostically differentiated children with typical language from those with SLI with an extremely high degree of likelihood.

It may be that the relationship between procedural memory and language that was observed in the current study differed from that of prior studies because statistical word learning was used to assess procedural memory in contrast to SRT or artificial grammar learning tasks and the focus was on lexical aspects of language as opposed to grammatical morphology or syntax. As in prior studies, in the current study, statistical word learning performance for the adolescents with SLI did not different from chance nor was it linked to any behavioral measure of lexical processing or cortical response. In contrast, for the CA controls, statistical word learning performance was correlated with speed of processing where better statistical word learning ability was linked to faster in detecting high-frequency words from nonwords. Notably, similar to the adolescents with SLI, statistical word learning was not linked to the modulation of the cortical response over any region for the CA controls. Given that there appears to be a linear relationship between better statistical word learning ability and vocabulary acquisition in infants, and to expressive and receptive vocabulary and speed of lexical processing in children with and without SLI, it may be that SWL abilities should be viewed as a continuum in a manner similar to hearing acuity/loss. Specifically, it may be that the ability to implicitly track information within the stream of speech is a fundamental mechanism that infants and children *will use* to acquire words in their lexicon if available. If, however, a child’s ability to implicitly track the probability of occurrence between and across a sequence of syllables or phonemes falls below some critical threshold, the child may be forced to rely on alternative compensatory strategies to acquire words in their lexicon.

A strength of the current study was the documented history of language abilities/deficits, where the participants’ group status as SLI or TD was determined at age 7;0 and then assessed thereafter until adolescence. Although the results from this study strongly suggest a relationship between impaired procedural memory and atypical brain organization for both lexical-phonological and lexical-semantic processing for the adolescents with SLI, the study was limited in identifying any direct link between impaired procedural memory and language processing due to at chance performance on the statistical word-learning task. Further, although knowledge of the language history of the participants was a strength, the attrition rate and subsequent small sample size that was available for the current study may have precluded the detection of a direct link between the cortical dynamics of lexical processing and procedural memory in either the SLI or CA groups. By tapping into the participant’s real-world knowledge, The Yes/No questions on the Competing Language Processing Task was used as an in direct measure of declarative memory. The strength of this approach was that the task was free of the verbal processing demands inherent in more traditional declarative memory tasks. It is possible, however, that better measures of declarative memory would highlight the compensatory reliance on this system, both in the behavioral performance and cortical responses of the adolescents with SLI.

Several additional substantive issues and limitations warrant further discussion. First, the findings from this study are based on group data and grand-average waveforms. Brown and colleagues [32] and Berglund-Barraza and colleagues [69] have both argued that group data obscures important differences in brain function in SLI. It is possible that examining the data at the group level in this study also obscured important individual differences in the pattern of cortical response on the part of the adolescents with SLI. Future studies using single-subject designs may be more illuminating with respect to the link between impaired procedural memory and the compensatory role of declarative memory and language acquisition in children with SLI. Similarly, the small number of participants in both the SLI and CA groups may have limited detection of more nuanced effects. An additional limitation of this study was the use of mean amplitude and animated topography as measures to identify potentially atypical patterns of cortical activation in SLI. Other methods such as time/frequency analysis of the EEG and/or MEG might prove more effective in characterizing individual differences in the pattern of dynamic cortical activity associated with language processing in adolescents with a history of SLI. Despite these limitations, the findings from this study support aspects of the PDH account of SLI and suggest that poor statistical word learning abilities may disrupt the acquisition and real-time processing of language at the lexical level in adolescents with SLI. The results from this study also highlight the small but growing body of work that suggests that while the behavioral performance of adolescents with SLI may overlap with that of their normal language peers, the cortical networks underlying this apparently "normal" performance may differ qualitatively from those of neurotypicals.

## Conclusions

The current study examined the neural correlates of lexical-phonological and lexical-semantic processing based on the predictions of the PDH in a group of adolescents with persistent SLI and a group of chronologically age-matched (CA) normal controls. The adolescents with SLI demonstrated intact declarative memory but impaired procedural memory. Based on behavioral performance, semantic conceptual knowledge also was intact in the adolescents with SLI whereas lexical phonological processing was impaired. Despite similar behavioral performance, the adolescents with SLI exhibit atypical cortical activation to the modulation of both semantic congruency and word frequency relative to CA controls. Based on the findings from this study, future studies examining the link between procedural memory and language in both children with and with SLI may need to address the different types of tasks used to assess procedural memory and the role the procedural memory plays in the acquisition and use of lexical and morphosyntactic aspects of the language system. The findings from this study also suggest that the link between procedural memory and language in both children with and without SLI may be more nuanced than that predicted by the PDH account and that behavioral performance and measures of cortical activation for both procedural and declarative memory and language need to be examined together. Finally, in future studies, it may be advantageous to treat procedural memory as a continuum where there is a critical threshold of ability, above which the child can leverage their procedural memory system to acquire language but below which the child may be forced to rely on alternative compensatory language acquisition strategies.

## Data Availability

The datasets used and/or analyzed during the current study are available from the corresponding author on reasonable request.

## Abbreviations

aMEG: Anatomically constrained magnetoencephalography
CA: Chronologically age-matched typical controls
CCV: Consonant consonant vowel
CLPT: Competing Language Processing Task
CV: Consonant vowel
Cz: Vertex reference
DLD: Developmental language disorder
EEG: Electroencephalography
EGI: Electrical Geodesics Inc.
ELAN: Early left anterior negativity
EpiSLI: Epidemiologic study of specific language impairment
ERP: Evoked response potential
HF: High-frequency
LF: Low-frequency
MEG: Magnetoencephalography
OAR: Ocular artifact removal
PCND: The Sand Diego Project in Cognitive and Neural Development
PDH: Procedural deficit hypothesis
ROI: Region of interest
SLI: Specific language impairment
SRT: Serial reaction time

## Footnotes

1 Arcsine transformed data *F* (1, 26) = 4.29, *p* = .05. Performance on 2AFC tasks is often measured via *d′* or *A′*, however, because performance was at ceiling for 11:28 participants (i.e., 100% probability hit rate of 1/false alarm rates of 0) it was not mathematically possible to calculate the area under the ROC curve and compared *A′* for the two groups.

2 Although *A′* has been reported in the literature [55] there is an error in the equation. The corrected *A* calculation is reported here [56].
